# Systematic Comparison of Heatmapping Techniques in Deep Learning in the Context of Diabetic Retinopathy Lesion Detection

**DOI:** 10.1167/tvst.9.2.64

**Published:** 2020-12-29

**Authors:** Toon Van Craenendonck, Bart Elen, Nele Gerrits, Patrick De Boever

**Affiliations:** 1 VITO NV, Unit Health, Mol, Belgium; 2 Hasselt University, Hasselt, Belgium; 3 University of Antwerp, Wilrijk, Belgium

**Keywords:** deep learning, heatmap, explainability, diabetic retinopathy

## Abstract

**Purpose:**

Heatmapping techniques can support explainability of deep learning (DL) predictions in medical image analysis. However, individual techniques have been mainly applied in a descriptive way without an objective and systematic evaluation. We investigated comparative performances using diabetic retinopathy lesion detection as a benchmark task.

**Methods:**

The Indian Diabetic Retinopathy Image Dataset (IDRiD) publicly available database contains fundus images of diabetes patients with pixel level annotations of diabetic retinopathy (DR) lesions, the ground truth for this study. Three in advance trained DL models (ResNet50, VGG16 or InceptionV3) were used for DR detection in these images. Next, explainability was visualized with each of the 10 most used heatmapping techniques. The quantitative correspondence between the output of a heatmap and the ground truth was evaluated with the Explainability Consistency Score (ECS), a metric between 0 and 1, developed for this comparative task.

**Results:**

In case of the overall DR lesions detection, the ECS ranged from 0.21 to 0.51 for all model/heatmapping combinations. The highest score was for VGG16+Grad-CAM (ECS = 0.51; 95% confidence interval [CI]: [0.46; 0.55]). For individual lesions, VGG16+Grad-CAM performed best on hemorrhages and hard exudates. ResNet50+SmoothGrad performed best for soft exudates and ResNet50+Guided Backpropagation performed best for microaneurysms.

**Conclusions:**

Our empirical evaluation on the IDRiD database demonstrated that the combination DL model/heatmapping affects explainability when considering common DR lesions. Our approach found considerable disagreement between regions highlighted by heatmaps and expert annotations.

**Translational Relevance:**

We warrant a more systematic investigation and analysis of heatmaps for reliable explanation of image-based predictions of deep learning models.

## Introduction

With deep learning (DL) we can now achieve excellent diagnostic performance on a wide range of medical image analysis tasks.^1^^–^^3^ However, the use of DL in clinical decision-making is still challenging and involves demonstrating the clinical utility of the algorithm, obtaining regulatory approval, and building trust and approval of the medical practitioner and patient. In this context, one of the tasks is to explain how and why a DL algorithm, which is often conceived as a black box model, makes a particular prediction.^4^ A wide range of heatmapping techniques has been introduced to produce visual maps to highlight regions in the image that contribute most to the prediction and thus explain the algorithm's decision.^5^^–^^7^

Being able to explain model decisions is important for a number of reasons. First, heatmaps build trust in the model when they corroborate clinically relevant features.^4^^,^^5^ Second, they can expose failure modes or hidden biases and suggest how models can be improved. For example, Winkler and colleagues^8^ inspect heatmaps and find that surgical skin markings in dermoscopic images significantly influence deep learning predictions. Third, experts can use the explanations to discover novel knowledge. For example, Poplin and colleagues^9^ report that age and sex can be predicted from retinal images. Ophthalmologists inspect blindly the heatmaps of 100 random retina images and identify for the first time that models trained to predict age focus on blood vessels and that models trained to predict sex highlight the optic disc, vessels, and macula. Intrigued by this finding Yamashita et al.^10^ discover that human assessed features such as optic disc ovality ratio, artery trajectory, and supratemporal retinal artery angle play a role in the DL models that predict sex.

Diabetic retinopathy (DR) is a common complication of diabetes and the leading cause of blindness among working age adults around the world.^11^^,^^12^ The presence of lesions such as microaneurysms, hemorrhages, hard exudates, venous beading, and other microvascular abnormalities in fundus images are used by human experts to score DR stage and disease severity. Several deep learning studies for DR staging have used heatmaps to explain model predictions.^13^^–^^16^ However, none of them quantify the performance of the heatmapping. Studies typically apply a single technique on a small selection of images to support visually DL explainability. However, it is reported that results vary significantly between images and methods.^17^^,^^18^

In this article we present a systematic and objective comparison of heatmapping techniques for DR. Popular deep learning network architectures (VGG16, InceptionV3 and ResNet50) trained to detect DR stage were combined systematically with each of 10 heatmapping techniques that are commonly reported in literature for visualization of DL output in the context of medical image analysis. Heatmaps were compared with common DR lesions in the images that were annotated by human experts. Comparisons were done with a newly developed metric, the Explainability Consistency Score (ECS).

This score quantifies how well the regions highlighted by the heatmapping technique match the regions marked independently by humans. The aim of the study was to investigate which heatmapping techniques are best able to detect regions of interest relevant for DR classification relative to a ground truth and to determine the impact of the DL network architecture used for DR classification.

## Material and Methods

### Deep Learning Models

Three deep learning models for diabetic retinopathy detection were trained and validated on the EyePACS KaggleDR dataset.^19^^,^^20^ This dataset contains 88704 images, which were randomly split in a training set of 68,074 images, a validation set of 10,000 images and a test set of 10,000 images. Patient level splits were maintained. All images were rescaled to 448 × 448 pixels. After rescaling, local average color was subtracted and mapped to 50% gray, and images were clipped to 90% size to remove boundary effects.^21^ Image augmentation was applied by rotating, shifting, and flipping images, as well as by applying zooms. We implemented versions of VGG16,^22^ InceptionV3,^23^ and ResNet50.^24^

The fully connected top layer was replaced with the following sequence of layers for all architectures: a global average pooling layer, two repetitions of a dense layer followed by a dropout layer, and finally a layer with only a single output with sigmoid activation function. A variant of the mean squared error that is tailored to ordinal classification was used as loss function for training.^25^ Models were evaluated using the quadratic weighted kappa, a modification to Cohen's kappa, that allows partial agreement.^26^ The weighted kappa allows the use of weighting schemes to take into account the closeness of agreement between categories, which is very suitable in case of using ordinal or ranked grades. The metric varies from 0, meaning random agreement, and 1, meaning complete agreement between raters. The quadratic weighted kappa was the reference metric in the Kaggle EyePacs challenge of 2016^20^ and since then it has been used frequently in publications evaluating deep learning for scoring DR severity from fundus pictures.^27^^–^^29^

The InceptionV3 model attained a quadratic weighted kappa of 0.82, and the ResNet50 and VGG16 models both had a quadratic weighted kappa of 0.79 on the test set. These scores are in the range of the top results attained in the KaggleDR competition.^20^ Further boost in performance was not considered for the current publication because the focus is on a comparative evaluation of explainability methods rather than deep learning prediction optimization.

### The IDRiD Dataset

The trained DL models were used as baseline for evaluating and comparing the different heatmapping techniques. These experiments were performed on the Indian Diabetic Retinopathy Image Dataset (IDRiD), which contains 81 images of DR patients from an Indian population with pixel level annotations.^30^

**Figure 1.**
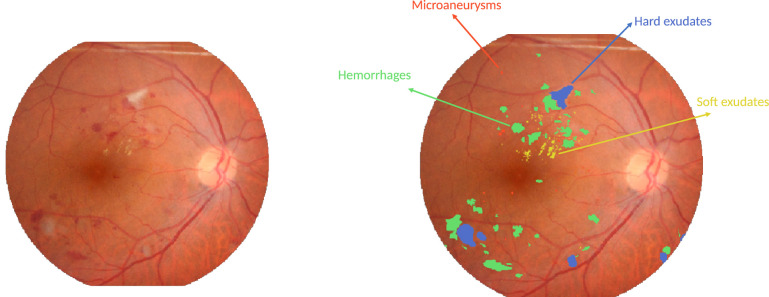
Example expert segmentation. *Left:* image from the IDRiD dataset; *right:* expert segmentation.

All subjects underwent mydriasis before collection of 50° field-of-view images with the Kowa VX-10a digital fundus camera. Pixel level annotations were done and reviewed by two retinal specialists. Four types of lesions were annotated: hemorrhages, microaneurysms, soft exudates, and hard exudates. Figure 1 shows an example of such an expert segmentation.

### Heatmapping Techniques

The 81 IDRiD images were analyzed with each of the three in advance trained deep learning models. None of the IDRiD images were used to train the DR scoring DL model. The IDRiD set was split into a tuning set of 54 images and a test set of 27 images. Heatmaps were generated for the 54 images using the 10 heatmapping techniques in Table 1.^4^^,^^6^^,^^27^^–^^33^ The test set was used for testing possible overfitting (see Results section). For Grad-CAM the implementation in the keras-vis library was used.^31^ In Grad-CAM we can visualize the filters of any convolutional layer. We tested every layer in the network for all tuning images and chose the one producing the best results on average. For the Gradients method, DeconvNet, Guided Backpropagation, Integrated Gradients, Input*Gradients, and Layerwise Relevance Propagation (LRP) the iNNvestigate library was used.^32^ We made custom implementations of SmoothGrad, SmoothGrad-Squared,^33^ and VarGrad,^33^ which rely on the iNNvestigate library to compute the gradients.^32^ LRP can be applied using several variants and parameter configurations. We experimented with 16 configurations (listed in Supplementary Information A) and selected the one that produced the best results in the training set for each architecture. This was LRP-Alpha2Beta1-IgnoreBias for VGG16, LRP-Alpha1Beta0-IgnoreBias for ResNet50 and LRP-Z for InceptionV3. Heatmapping techniques (except Grad-CAM) produce a heatmap with three channels and the output was converted to a single channel heatmap by taking the maximum over the channels.

**Table.** Heatmapping Techniques Used for Explainability in Deep Learning Models for Diabetic Retinopathy Prediction From Fundus Images

Heatmapping Technique	Reference
Gradients	Simonyan et al.^22^
Integrated gradients	Sundararajan et al.^35^
Input * gradient	Shrikumar, Greenside, & Kundaje, 2017^40^
Guided backpropagation	Springerberger et al.^6^
LRP	Bach et al., 2015^41^
Grad-CAM	Selvaraju et al.^4^
DeconvNet	Zeiler & Fergus, 2014^42^
SmoothGrad	Smilkov, Thorat, Kim, Viegas, & Wattenberg, 2017^43^
SmoothGrad-Squared	Hooker et al.^33^
VarGrad	Hooker et al.^33^

### The Explainability Consistency Score

We define the ECS to compare the heatmaps to ground truth lesion expert segmentations.

The computation of the ECS consists of two steps: the expert segmentation and the computer-generated heatmap are discretized (Step 1), and afterwards the agreement between the two discretized maps is computed (Step 2).

#### Step 1: Discretization

**Figure 2.**
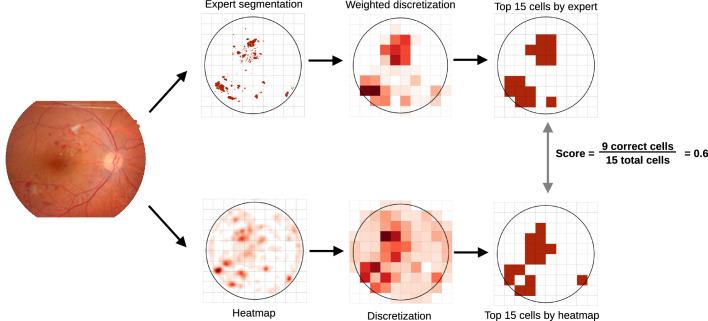
Illustration of the ECS calculation for a 10 × 10 grid.

The expert segmentations were discretized by overlaying the fundus image with a grid and counting the number of lesion pixels in each cell. An example of a 10 × 10 grid overlaid to one of the images in the IDRiD dataset is given in Figure 2.

The pixel count was weighted by the lesion types to deal with severe pixel count imbalance between lesion types: in the expert segmentations of the IDRiD dataset 1% of the pixels indicated as lesions by the experts correspond to microaneurysms, 8% correspond to soft exudates, 44% correspond to hard exudates and 47% correspond to hemorrhages. Not taking this imbalance into account would lead to microaneurysms barely contributing to the agreement score, although they are crucial for identifying early stages of DR diagnosis.^34^ We denote the segmentation of hemorrhages as a matrix as $HE\in R^{N\times N}$, with *HE*_*i*,*j*_ = 1 if the expert indicated the presence of a hemorrhage at that pixel and *HE*_*i*,*j*_ = 0 otherwise. Similarly, *MA* describes microaneurysms, *EX* hard exudates and *SE* soft exudates. The discretized expert segmentation *DE* combines these four matrices into one by taking weighted sums of these segmentation matrices in grid cells. For an *S* × *S* grid we computed *DE*_*i*,*j*_ for *i*, *j* ∈ 1...*S* of the discretized expert segmentation as follows:

$$\begin{array}{c} \begin{array}{c} DE_{i,j}=w_{HE}\sum_{p\;=\;id}^{\left(i+1\right)d-1}\sum_{q\;=\;jd}^{\left(j+1\right)d-1}HE_{p,q}+w_{MA}\sum_{p\;=\;id}^{\left(i+1\right)d-1}\sum_{q\;=\;jd}^{\left(j+1\right)d-1}MA_{p,q}\\ \;+\;w_{SE}\sum_{p\;=\;id}^{\left(i+1\right)d-1}\sum_{q\;=\;jd}^{\left(j+1\right)d-1}SE_{p,q}+w_{EX}\sum_{p\;=\;id}^{\left(i+1\right)d-1}\sum_{q\;=\;jd}^{\left(j+1\right)d-1}EX_{p,q}\;\; \end{array} \end{array}$$

with $d=\frac{N}{S}$ the width/height of a cell, and *w_HE_*, *w_MA_*, *w_SE_*, and *w_EX_* the weights for hemorrhages, microaneurysms, soft exudates, and hard exudates. We determined the weight for each lesion type by its pixel count over all training images, relative to the pixel count of the most frequently occurring lesion type. This led to the following weights for the IDRiD dataset: *w_HE_* = 1,  *w_EX_* = 1.05,  *w_SE_* = 5.77 and *w_MA_* = 46.97.

The heatmap discretization was computed in a similar manner but without weighting, as heatmaps do not distinguish between different lesion types. For a heatmap *H* ∈ *R*^*N* × *xN*^ entry *DH*_*i*,*j*_ for i,j ∈ 1..S of the discretized heatmap *DH* ∈ *R*^*S* × *xS*^ was computed as follows:

$$\begin{array}{c} DH_{i,j}=\sum_{p\;=\;id}^{\left(i+1\right)d-1}\sum_{q\;=\;jd}^{\left(j+1\right)d-1}H_{p,q}\; \end{array}$$

#### Step 2: Calculation of Agreement Between Discretized Maps

The agreement between the discretized expert segmentation and the discretized heatmap was computed:

$$\begin{array}{c} \begin{array}{c} ECS\left(DE,DH\right)=\frac{\left|top\_k\_cells\left(DE,K\right)\cap top\_k\_cells\left(DH,K\right)\right|}{\min\left(K,n\_nonzero\_elements\left(DE\right)\right)} \end{array} \end{array}$$

*top_k_cells* is a helper function that returns the row and column indexes of the *K* cells with the highest value. The *n_nonzero_elements* return the number of nonzero elements in a matrix. We divide by *K* (or the number of nonzero elements in the discretized expert segmentation if this is smaller than *K*) such that ECS our score always takes values in [0, 1] and can be interpreted as a percentage. We tested the sensitivity to the selection of the *K* parameter by experimenting with *K* = 10,  15 and 20. The choice of this parameter did not affect our main outcomes. Figure 2 summarizes the computation of the ECS score.

## Results

**Figure 3.**
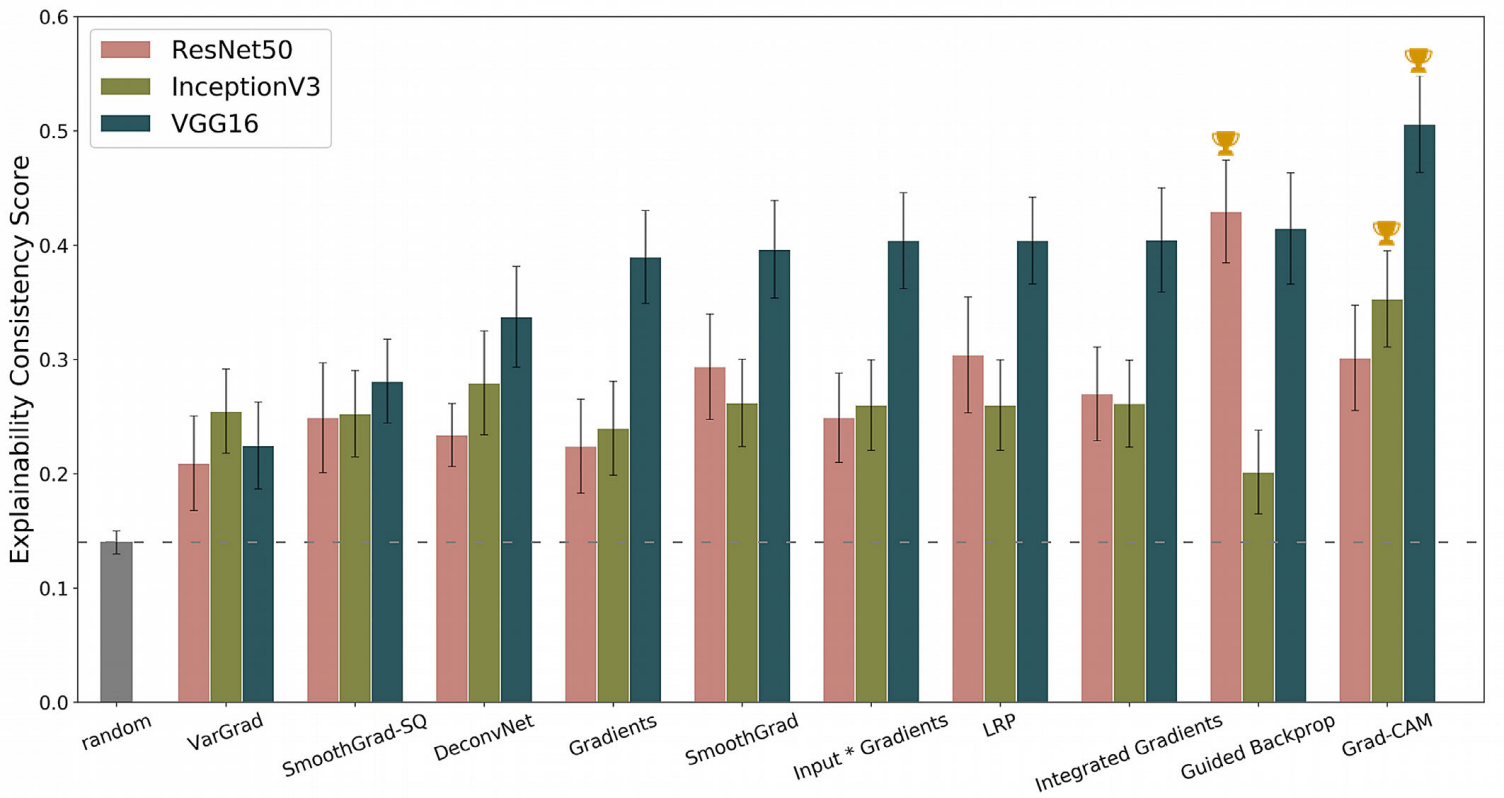
Average ECS for the three different deep learning models and each of the 10 heatmapping techniques. Results are obtained with a tuning subset of the IDRiD image dataset (*n* = 54). The top three combinations are marked by the *cup* on top of the *bars*.

Figure 3 presents the average values of the Explainability Consistency Score (ECS) for the IDRiD tuning set of 54 fundus images (See Supplementary Information B for detailed numerical results). The scores range from 0.21 to 0.51, with 0 meaning no overlap between pixels marked by the heatmap and pixels marked by experts during lesion annotation and 1 referring to complete agreement between both. The top three combinations are indicated in Figure 3, with VGG16 + Grad-CAM overall highest (ECS = 0.51; 95% confidence interval [CI]: [0.46;0.55]). The selected Grad-CAM configuration was the one in which the pixels responsible for the activation of the filters of the first convolutional layer of the last convolutional block are visualized. The VGG16 architecture compared favorably to the other architectures in combination with most heatmapping techniques, that is, approximately 0.05 to 0.1 higher ECS or 5% to 10% more correspondence with human annotations. The gray bar in Figure 3 and dashed line refer to the score obtained by using a heatmap that attributes importance randomly. All combinations significantly outperform this baseline. Values of ECS between 0.21 to 0.51 illustrate that the choice of the architecture and heatmapping technique impact how much visual overlap there will be with the actual DR lesion segmentation done by experts. Possible overfitting was investigated by computing the scores for the 27 images in the IDRiD test set for the VGG16+Grad-CAM combination. The average ECS score on the tuning set was 0.51 (95% CI: [0.46; 0.55]) and the test set score was 0.48 (95% CI: [0.44; 0.53]). This is only a modest decrease, which is suggestive for the fact that our selection procedure was not overfitting. We also investigated whether the performance of the ECS score varied according to the DR severity class using our IDRiD tuning set. Grade 2 images 2 (n = 18) had an ECS score of 0.47 (95% CI: [0.43; 0.58]), Grade 3 (n = 16) and 4 (n = 19) images had an ECS score of 0.54 (95% CI: [0.43; 0.58]) and 0.50 (95% CI: [0.43; 0.58]), respectively. Only one image of Grade 1 was available in the IDRiD dataset. Data suggests no bias according to severity class, but the available number of images is low for a conclusive evaluation.

We present the ECS score with a 10 × 10 grid, taking the top 15 cells with the highest values in the discretized maps into account for computing the score (i.e., *S* = 10 and *K* = 15 ). Results for additional experiments can be found in Supplementary Information C for reference. A 10 × 10 grid was selected because it has the granularity to indicate potentially interesting regions to an ophthalmologist and it can deal with the outputs produced by the different heatmapping techniques.

The heatmaps produced by Grad-CAM, for example, were very different from those produced by Guided Backpropagation, and discretization in a 10 × 10 grid allows our score to be sensitive to the regions that these heatmaps indicate instead of their pixel level appearances.

**Figure 4.**
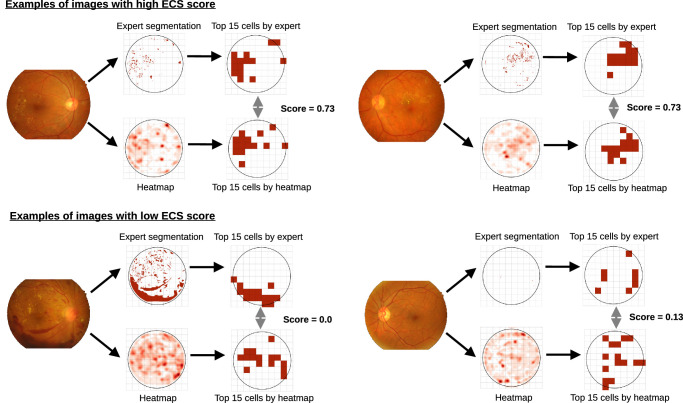
Examples of images with high and low ECS scores. Two heatmaps generated by VGG16+Grad-CAM with the highest score on the IDRiD training set (*top row*), and two heatmaps with the lowest score (*bottom row*).

The top row in Figure 4 illustrates the ground truth and heatmaps for the two images with the highest scores in the IDRiD training set. For both images, Grad-CAM successfully identified 11 of the top 15 cells in the expert segmentation, which correspond to exudates and hemorrhages. The two images with the lowest scores are shown in the bottom row in Figure 4. The left fundus shows that the model did not attribute importance to the large bleeding, resulting in a low ECS score. In the right fundus on the bottom row, there are only a few small lesions. The heatmap covers several of them, but also highlights several cells that are not marked by experts, which results in the low score.

**Figure 5.**
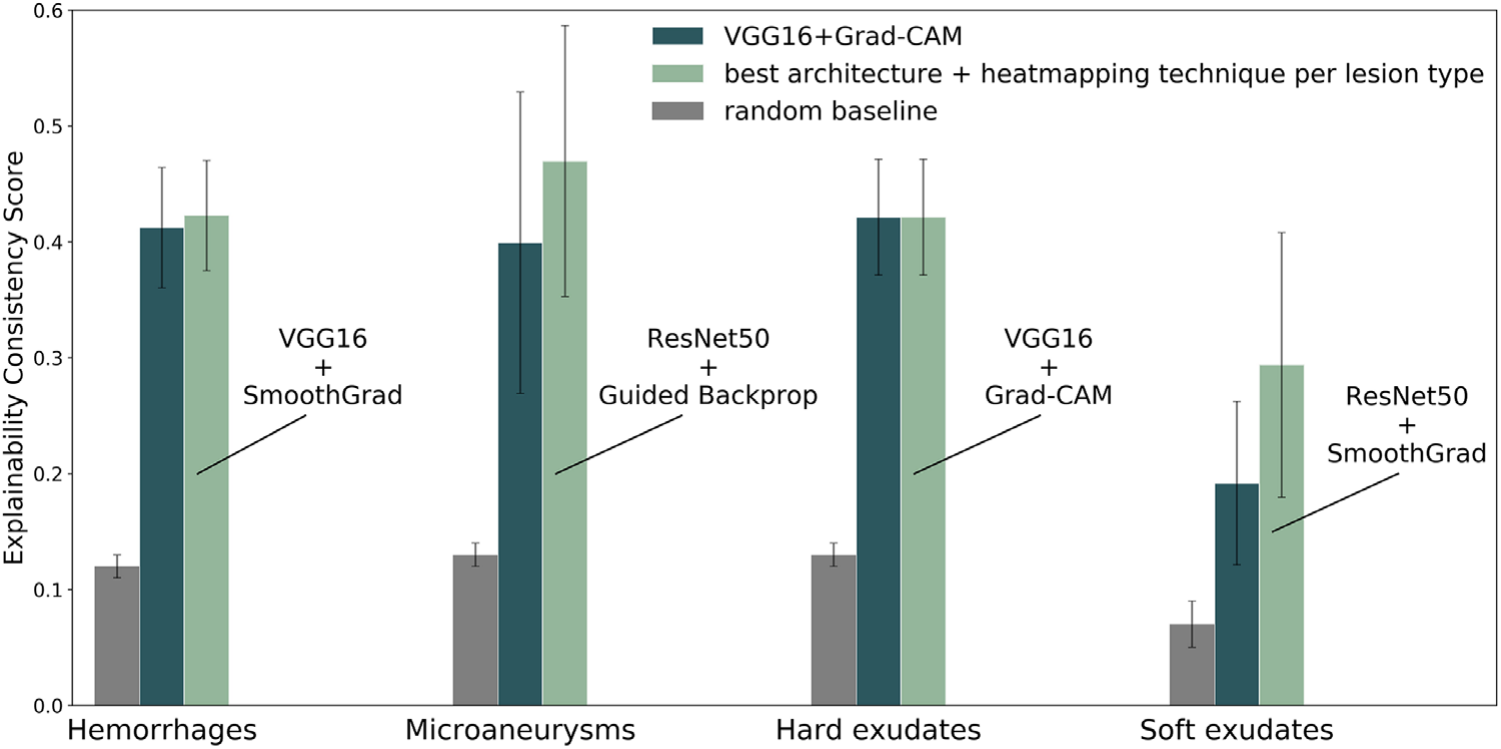
Average ECS scores computed separately for each lesion type. The results are shown for the random baseline, the overall best configuration (VGG16+Grad-CAM), and the configuration that scored best for this lesion type.

The ECS values in Figure 3 are computed by comparing model explanations to a weighted combination of expert segmentations of hard exudates, soft exudates, hemorrhages and microaneurysms. We also computed the ECS separately for the different lesion types to investigate whether there were large differences in performance between them. Figure 5 summarizes the results of these experiments. The supplement contains the figures and numerical data of the experiments for the DL models/heatmapping techniques for the individual lesions (Supplementary Information D and E). No weighting was performed for these computations: the discretized model explanation was compared to the discretized lesion segmentation for one specific lesion type. The ECS score varied significantly across lesion types. ECS was lowest for soft exudates (0.29 for the best architecture and heatmapping combination) and highest for microaneurysms (0.42 for the best combination). The overall best combination, VGG16+Grad-CAM, consistently performed well for all lesion types, but was not the best performing combination for each individual lesion type. For example, ResNet50+SmoothGrad performed best for soft exudates, and ResNet50+Guided Backpropagation performed best for microaneurysms.

## Discussion

This study presents a systematic and objective comparison between heatmapping techniques in deep learning (DL) using diabetic retinopathy lesion detection as a case study. Several studies on DL for DR detection have used a single heatmap to support their model predictions. Lam et al.^15^ generate heatmaps using patch-wise occlusion, Bellemo et al.^13^ use Integrated Gradients, and Sundararajan and colleagues^35^ and Gargeya and Leng^14^ use Grad-CAM.^4^ Heatmaps of a small selection of images are typically submitted for visual inspection. The result of this analysis is then used to support the fact that the AI model is trustworthy because it is looking at the expected locations. Sayres et al.^16^ generate heatmaps using Integrated Gradients and found that accuracy of the DR diagnosis improves when a trained grader is given the DL predicted DR grade and heatmap to make the DR diagnosis for moderate-or-worse DR. They found that providing heatmaps for cases with no DR can cause graders to overcall these cases but observed that this effect diminished over time as graders learned to use the heatmaps for guiding diagnosis. However, it is reported that heatmaps can differ significantly depending on the used technique. Hence, variation in the provided visual support may impact any diagnostic judgment.

In the context of general image classification tasks, studies have aimed to compare heatmapping techniques. Selvaraju et al.^4^ evaluate class discriminativeness of several methods based on human studies. Others have defined properties that a heatmapping method should satisfy, such as completeness, implementation invariance, sensitivity and input invariance.^35^^,^^36^ One can evaluate methods by verifying which properties they satisfy or construct new ones that satisfy certain properties. This does not necessarily provide the practitioner with enough guidance for selecting a method for an application because no method satisfies all properties and it is hard to determine which ones are most relevant for a specific application. Recently, Hooker et al. have proposed RemOve And Retrain (ROAR), which evaluates explainability by measuring how the accuracy of retrained models degrades as pixels that are estimated to be important are removed.^33^ According to their evaluation methodology, most of the heatmapping/explainability methods are not better than random designation of feature importance. They find that only VarGrad and SmoothGrad-Squared outperform the random baseline.

Our evaluation is focused on DR detection, whereas most prior work was performed with the ImageNet classification task.^37^ These tasks differ considerably, and we cannot expect conclusions drawn for ImageNet to transfer directly to our application. We are the first to do an objective and fair comparison of heatmapping techniques in the context of DR detection. A second difference to prior work is that we leverage expert segmentations. This allowed us to circumvent many of the problems that related studies face due to the absence of ground truth data. The pixel level expert annotations in the IDRiD dataset are separated by lesion type, and this enabled us to tailor the ECS metric to our application by weighting pixels differently according to their type. Most heatmapping methods outperformed significantly the random baseline in our experiments and thus contradict the suggestion of Hooker et al.^33^ that the majority of the methods are not better than random designation of feature importance. It is important to note that the evaluation strategies are different: whereas Hooker and colleagues^33^ evaluate interpretability by measuring how the accuracy of retrained models degrades as pixels that are estimated to be important are removed, we do it by comparing heatmaps to expert segmentations. Although most explainability methods significantly outperformed the random baseline, there is still considerable room for improvement. The best-performing configuration on average correctly identified 51% of the most important lesion cells on the IDRiD training dataset, indicating that there is still disagreement between regions highlighted by heatmaps and expert annotations. This could be because the DL model weighs in additional information for DR detection such as various blood vessel changes, which is not present in the IDRiD annotation. However, this was not further investigated.

The task of evaluating how well a heatmap matches an expert segmentation is like that of evaluating segmentations produced by image segmentation methods. Three key differences, however, render existing evaluation scores for segmentation (such as DICE) inadequate.^38^ First, pixel level agreements between heatmaps and expert segmentations are not relevant. Heatmaps must mark the right regions in the image, but pixel level segmentation accuracy is not their goal.

Second, existing scores have difficulties dealing with the highly different types of outputs that are generated by various heatmapping techniques. For example, a Grad-CAM heatmap typically marks larger regions as being important, while Integrated Gradients marks individual pixels: any pixel-based score will behave very differently for Grad-CAM and Integrated Gradients, obstructing a fair comparison between the two. Third, existing scores do not incorporate domain knowledge and treat every pixel equally. For example, a pixel labeled as a microaneurysm will have the same weight as a pixel labeled as a hemorrhage, which does not reflect the importance of microaneurysms in DR diagnosis: despite their small size in terms of pixel count, they strongly influence early level DR diagnosis.^34^ The ECS metric deals with the first two points by discretizing heatmaps and expert segmentations. The result of this step is that exact pixel locations and types of heatmap do not matter, but only the regions that they indicate as being important. The third point is addressed by weighting the pixels in the expert annotation by their lesion type, such that small lesions that are important for diagnosis contribute sufficiently in the ECS calculation.

A study limitation is that not all lesions that characterize DR are annotated in the IDRiD dataset. It contains expert segmentations of hard exudates, soft exudates, microaneurysms, and hemorrhages, but not for several other, less abundantly present, abnormalities that are associated with DR such as venous beading, intraretinal microvascular abnormalities (IRMAs), and neovascularization.^34^ This limitation could of course be alleviated by obtaining expert segmentations for all lesion types. Alternatively, it would be interesting to capture eye tracking data of ophthalmologists performing DR grading, similar to the work of Li et al. for glaucoma detection.^39^ We would expect such eye tracking data to cover all relevant lesions, and as such provide for a natural point of comparison for the produced heatmaps produced. A second limitation is the fact that the ECS score does not consider the lesion location. The latter can impact its role in DR diagnosis. For example, severe nonproliferative diabetic retinopathy is indicated by, among others, venous beading in at least two quadrants.^34^

## Conclusions

This work objectively evaluated heatmapping techniques in deep learning in the context of diabetic retinopathy. Heatmaps were compared to common DR lesions that were annotated at pixel level by expert graders. The proposed ECS tailored to DR scoring enabled a fair comparison between heatmaps and expert annotations. An empirical evaluation of three DL models for DR detection in combination with each of 10 heatmapping techniques is presented.

The best overall explainability results were reached with Grad-CAM and a VGG16 model. For the ResNet50 model, the best explainability results were obtained with Guided Backpropagation and for the InceptionV3 model this was Grad-CAM. We found considerable differences between heatmapping techniques when trying to identify relevant diabetic retinopathy lesions. We warrant a more systematic investigation and analysis of heatmaps for reliable explanation of image-based predictions of deep learning models.

## Supplementary Material

Supplement 1
