# Supplementary material for: Systematic Comparison of Heatmapping Techniques in Deep Learning in the Context of Diabetic Retinopathy Lesion Detection
Source: Transl Vis Sci Technol. 2020 Dec 29;9(2):64. doi: 10.1167/tvst.9.2.64 (PMC7774113; doi:10.1167/tvst.9.2.64)
Supplement: Supplement 1 [file tvst-9-2-64_s001.docx]

**Supplementary information**

**A)**

More information on the configurations for LRP can be found in the documentation at <https://innvestigate.readthedocs.io/>. We used the following configurations: LRP-ZPlusFast, LRP-SequentialPresetB, LRP-WSquare, LRP-ZPlus, LRP-Epsilon, LRP-Alpha2Beta1, LRP-SequentialPresetBFlat, LRP-Alpha1Beta0_ignorebias, LRP-SequentialPresetAFlat, LRP-Alpha2beta1_ignorebias, LRPFlat, LRP-Alpha1Beta0, LRP-Epsilon_ignorebias, LRP-Z_ignorebias, LRP-SequentialPresetA.

**B)**

|  | ResNet50 | InceptionV3 | VGG16 |
| --- | --- | --- | --- |
| Gradients | 0.22 (0.18, 0.27) | 0.24 (0.20, 0.28) | 0.39 (0.35, 0.43) |
| Input * Gradients | 0.25 (0.21, 0.29) | 0.26 (0.22, 0.30) | 0.40 (0.36, 0.45) |
| Integrated Gradients | 0.27 (0.23, 0.31) | 0.26 (0.22, 0.30) | 0.40 (0.36, 0.45) |
| Guided Backpropagation | 0.43 (0.38, 0.47) | 0.20 (0.16, 0.24) | 0.41 (0.37, 0.46) |
| LRP | 0.30 (0.25, 0.35) | 0.26 (0.22, 0.30) | 0.40 (0.37, 0.44) |
| Grad-CAM | 0.30 (0.26, 0.35) | 0.35 (0.31, 0.39) | **0.51 (0.46, 0.55)** |
| DeconvNet | 0.23 (0.21, 0.26) | 0.28 (0.23, 0.33) | 0.34 (0.29, 0.38) |
| SmoothGrad | 0.29 (0.25, 0.34) | 0.26 (0.22, 0.30) | 0.40 (0.35, 0.44) |
| SmoothGrad-SQ | 0.25 (0.20, 0.30) | 0.25 (0.21, 0.29) | 0.28 (0.24, 0.32) |
| VarGrad | 0.21 (0.17, 0.25) | 0.25 (0.22, 0.29) | 0.22 (0.19, 0.26) |
| Baseline | 0.13 | 0.13 | 0.13 |

*Table B.1 Average ECS on the IDRiD training set and 95% confidence intervals.*

**C)**

**
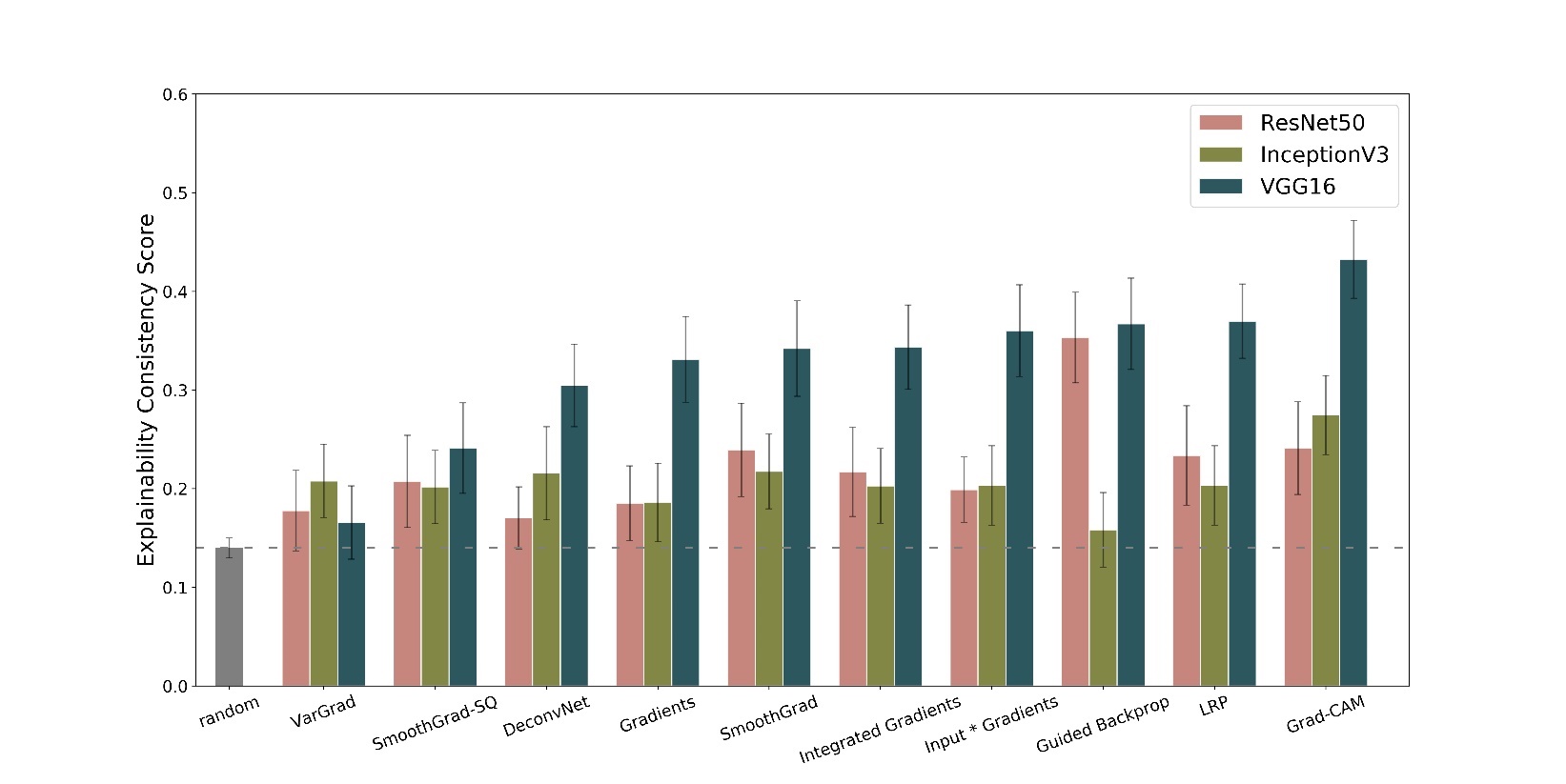
**

*Figure C.1 Average ECS scores for different DL models and heatmapping techniques for* $K=10$ *in the ECS calculation.*

**
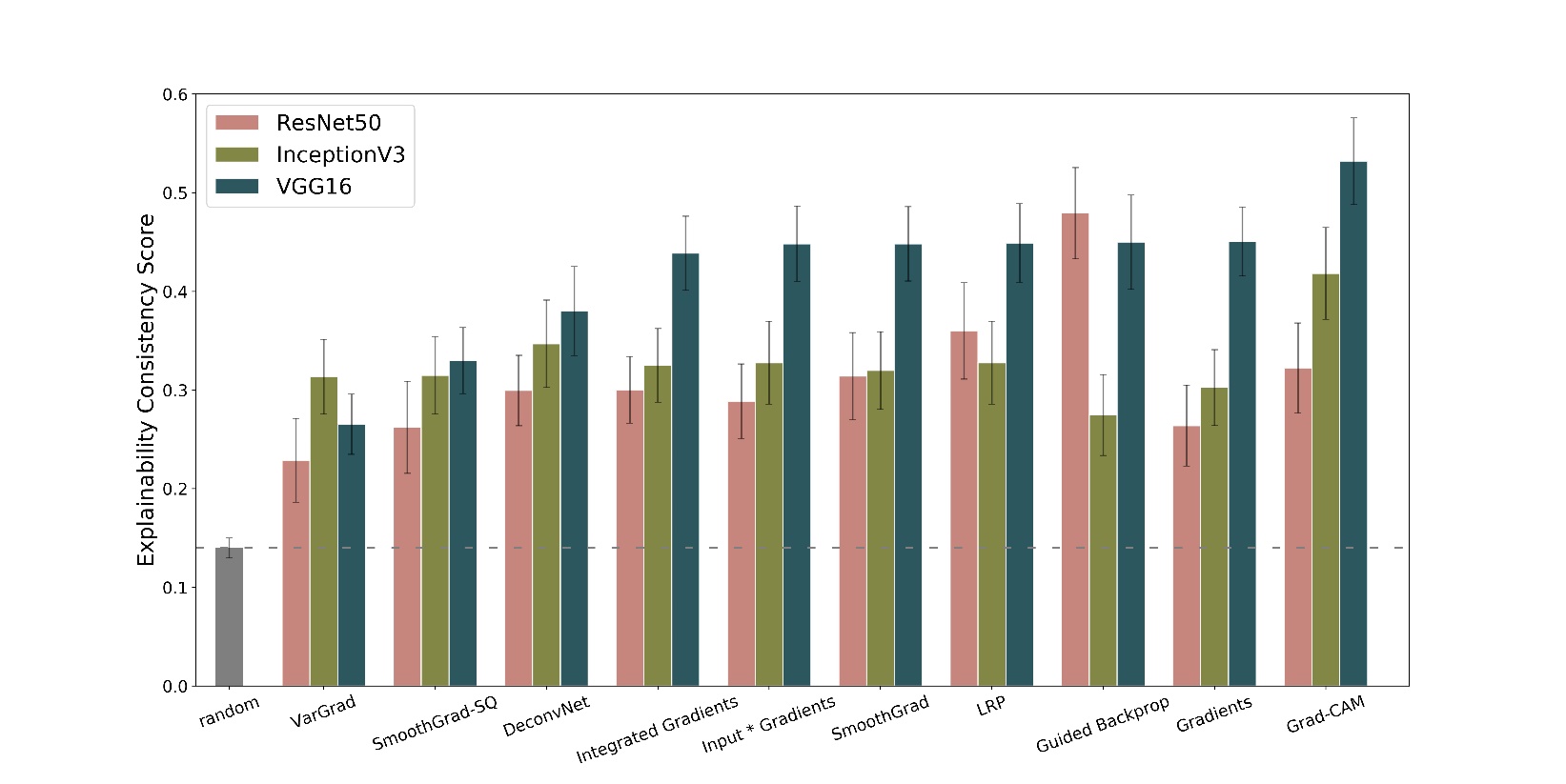
**

*Figure C.2 Average ECS scores for different DL models and heatmapping techniques for* $K=20$*in the ECS calculation.*

**D)**

*
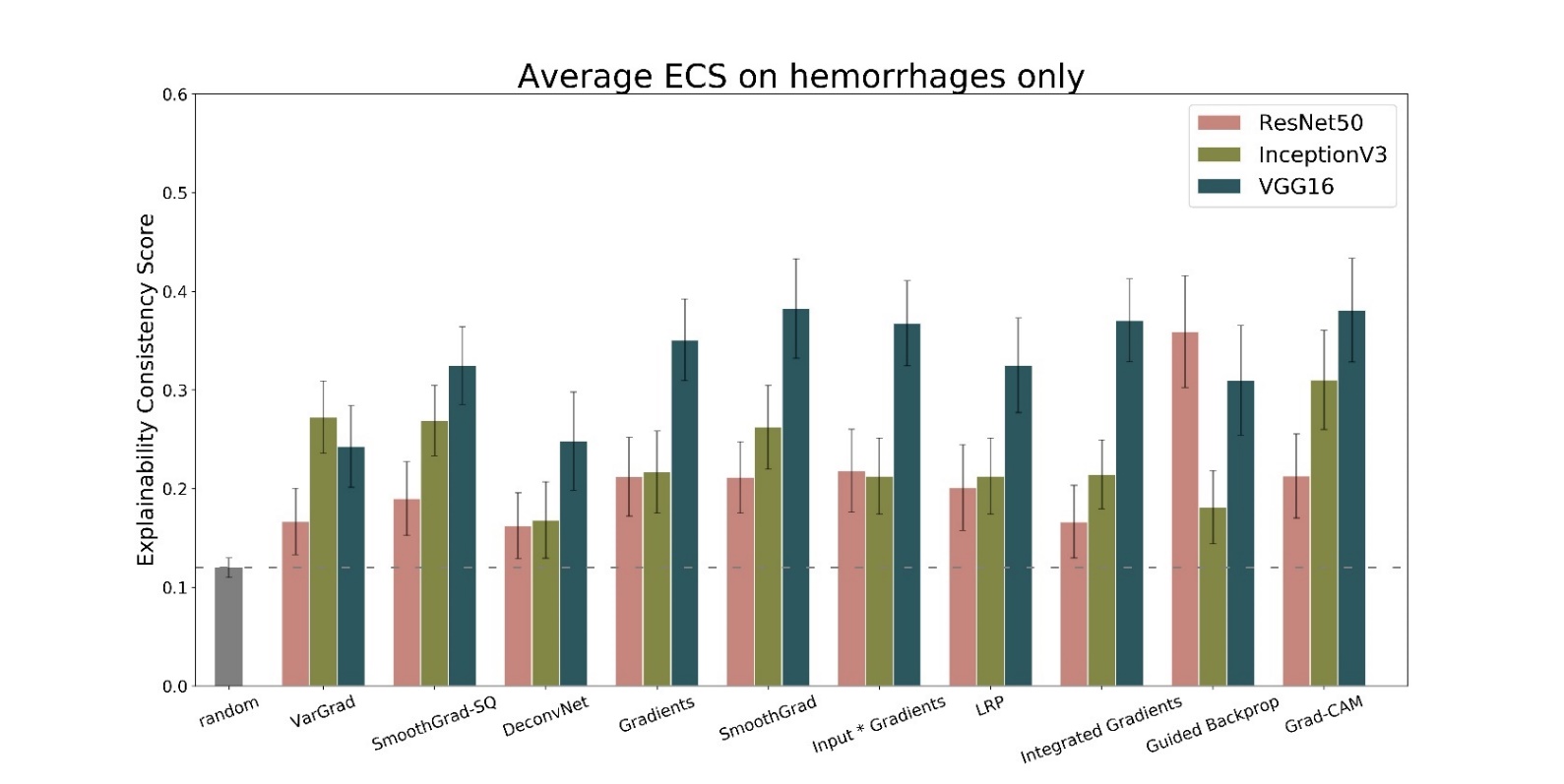
*

*Figure D.1 Average ECS scores for different DL models and heatmapping techniques, taking into account hemorrhages only. The scores are averaged over the 53 IDRiD training images that contain hemorrhages.*

*
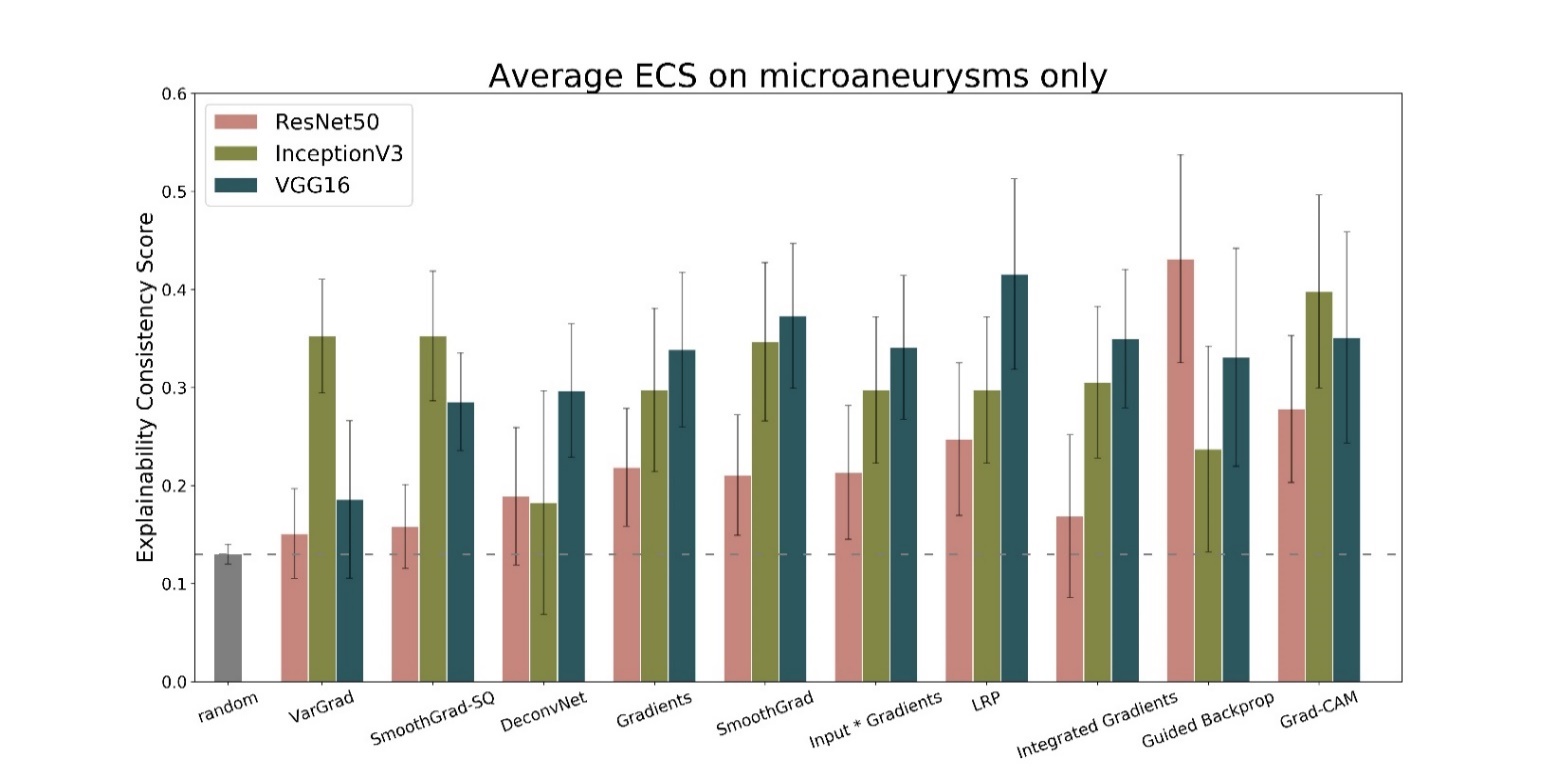
*

*Figure D.2 Average ECS scores for different DL models and heatmapping techniques, taking into account microaneurysms only. The scores are averaged over the 8 IDRiD training images that contain microaneurysms.*

**D)**

*
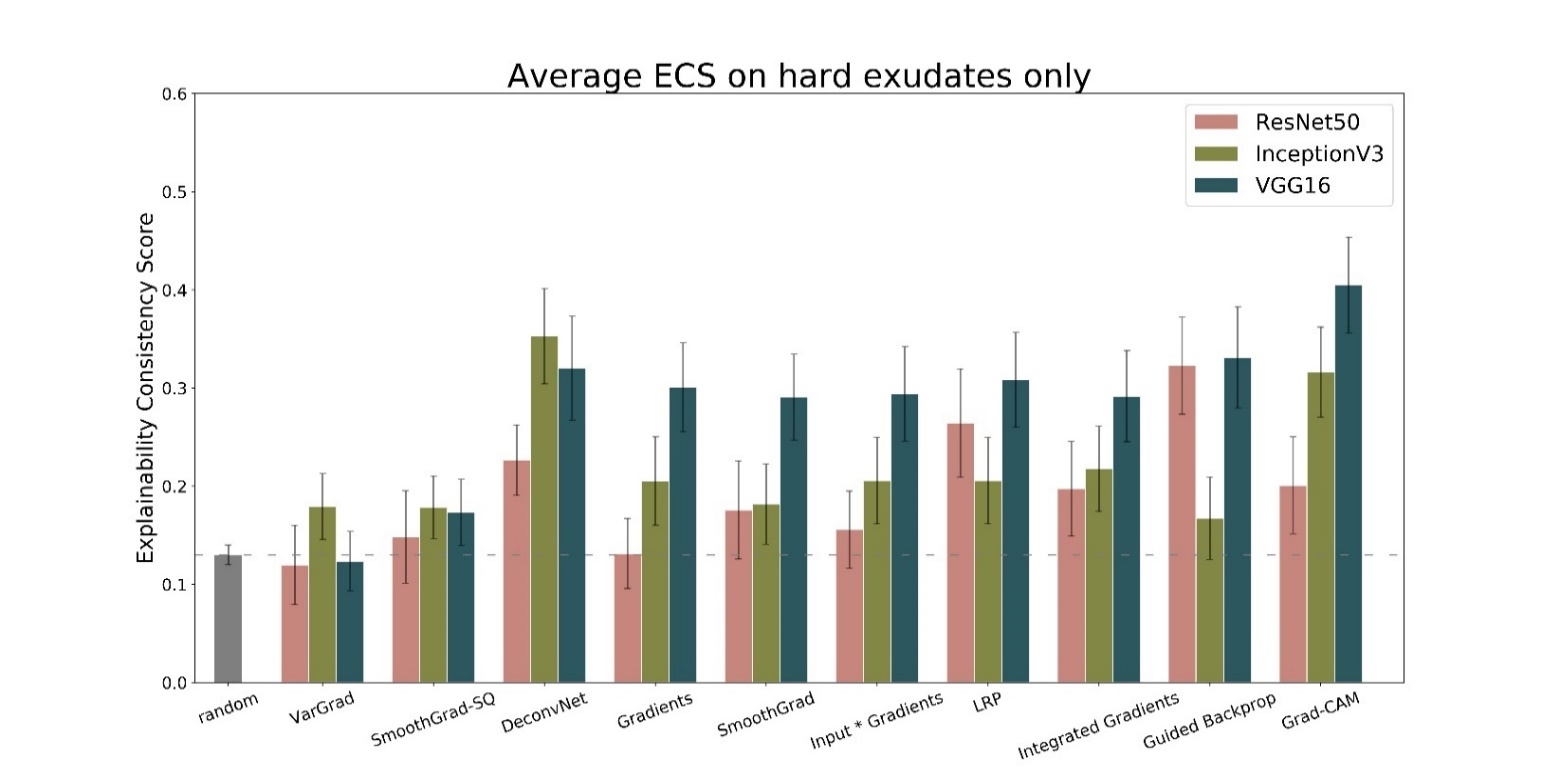
*

*Figure D.3 Average ECS scores for different DL models and heatmapping techniques, taking into account hard exdates only. The scores are averaged over the 53 IDRiD training images that contain hard exudates.*

*
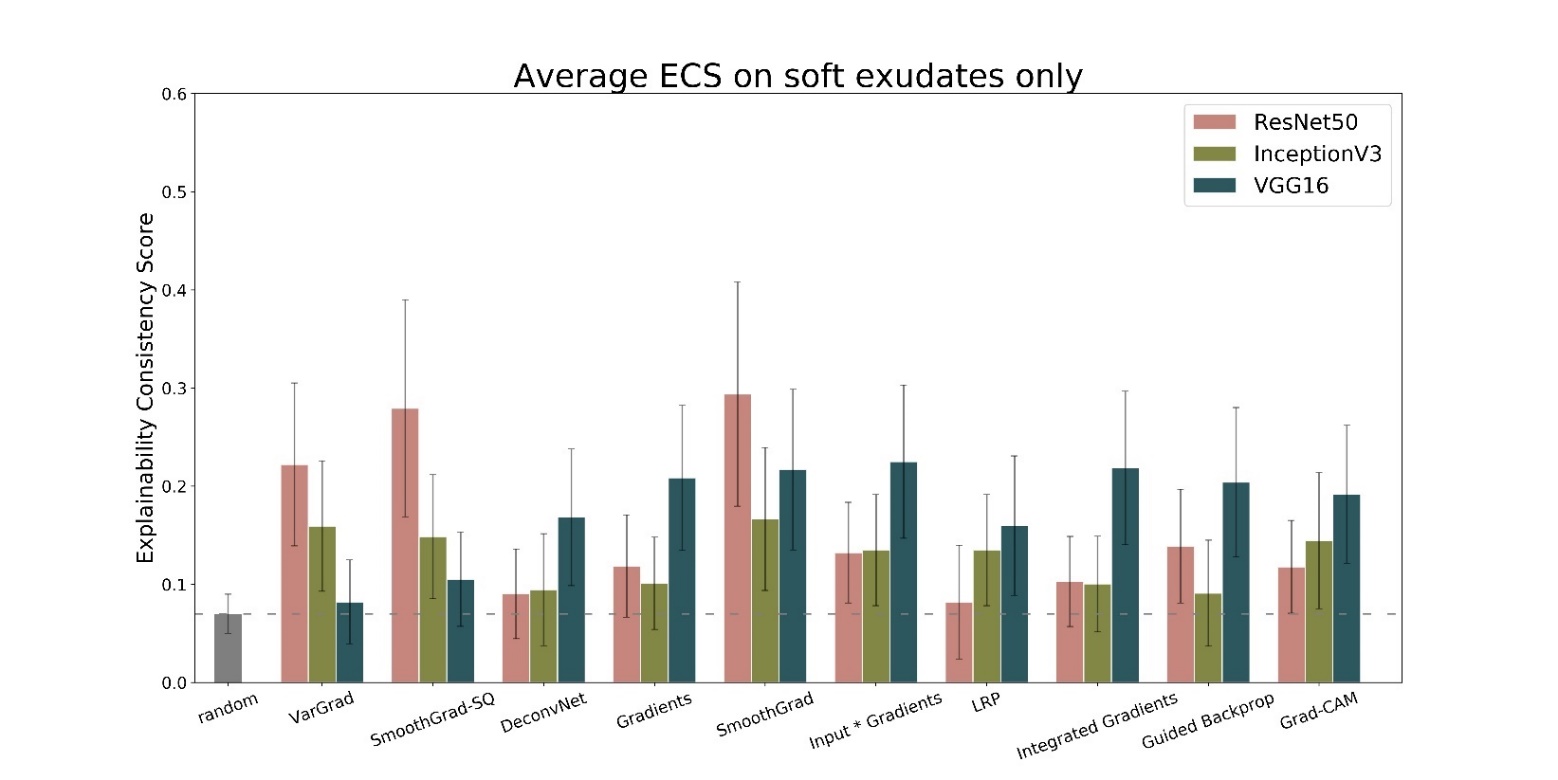
*

*Figure D.4 Average ECS scores for different DL models and heatmapping methods, taking into account soft exudates only. The scores are averaged over the 26 IDRiD training images that contain soft exudates.*

**E)**

|  | ResNet50 | InceptionV3 | VGG16 |
| --- | --- | --- | --- |
| Gradients | 0.13 (0.10, 0.17) | 0.21 (0.16, 0.25) | 0.30 (0.26, 0.35) |
| Input * Gradients | 0.16 (0.12, 0.20) | 0.21 (0.16, 0.25) | 0.29 (0.25, 0.34) |
| Integrated Gradients | 0.20 (0.15, 0.25) | 0.22 (0.17, 0.26) | 0.29 (0.25, 0.34) |
| Guided Backpropagation | 0.32 (0.27, 0.37) | 0.17 (0.13, 0.21) | 0.33 (0.28, 0.38) |
| LRP | 0.26 (0.21, 0.32) | 0.21 (0.16, 0.25) | 0.31 (0.26, 0.36) |
| Grad-CAM | 0.20 (0.15, 0.25) | 0.32 (0.27, 0.36) | 0.40 (0.36, 0.45) |
| DeconvNet | 0.23 (0.19, 0.26) | 0.35 (0.30, 0.40) | 0.32 (0.27, 0.37) |
| SmoothGrad | 0.18 (0.13, 0.23) | 0.18 (0.14, 0.22) | 0.29 (0.25, 0.33) |
| SmoothGrad-SQ | 0.15 (0.10, 0.20) | 0.18 (0.15, 0.21) | 0.17 (0.14, 0.21) |
| VarGrad | 0.12 (0.08, 0.16) | 0.18 (0.15, 0.21) | 0.12 (0.09, 0.15) |
| Baseline | 0.13 (0.12, 0.14) | 0.13 (0.12, 0.14) | 0.13 (0.12, 0.14) |

*Table E.1 Average ECS on the IDRiD training set and 95% confidence intervals, for hard exudates only.*

|  | ResNet50 | InceptionV3 | VGG16 |
| --- | --- | --- | --- |
| Gradients | 0.22 (0.16, 0.28) | 0.30 (0.21, 0.38) | 0.34 (0.26, 0.42) |
| Input * Gradients | 0.21 (0.15, 0.28) | 0.30 (0.22, 0.37) | 0.34 (0.27, 0.41) |
| Integrated Gradients | 0.17 (0.09, 0.25) | 0.31 (0.23, 0.38) | 0.35 (0.28, 0.42) |
| Guided Backpropagation | 0.43 (0.33, 0.54) | 0.24 (0.13, 0.34) | 0.33 (0.22, 0.44) |
| LRP | 0.25 (0.17, 0.33) | 0.30 (0.22, 0.37) | 0.42 (0.32, 0.51) |
| Grad-CAM | 0.28 (0.20, 0.35) | 0.40 (0.30, 0.50) | 0.35 (0.24, 0.46) |
| DeconvNet | 0.19 (0.12, 0.26) | 0.18 (0.07, 0.30) | 0.30 (0.23, 0.37) |
| SmoothGrad | 0.21 (0.15, 0.27) | 0.35 (0.27, 0.43) | 0.37 (0.30, 0.45) |
| SmoothGrad-SQ | 0.16 (0.12, 0.20) | 0.35 (0.29, 0.42) | 0.29 (0.24, 0.34) |
| VarGrad | 0.15 (0.11, 0.20) | 0.35 (0.29, 0.41) | 0.19 (0.11, 0.27) |
| Baseline | 0.13 (0.12, 0.14) | 0.13 (0.12, 0.14) | 0.13 (0.12, 0.14) |

*Table E.2 Average ECS on the IDRiD training set and 95% confidence intervals, for microaneurysms only.*

|  | ResNet50 | InceptionV3 | VGG16 |
| --- | --- | --- | --- |
| Gradients | 0.21 (0.17, 0.25) | 0.22 (0.18, 0.26) | 0.35 (0.31, 0.39) |
| Input * Gradients | 0.22 (0.18, 0.26) | 0.21 (0.17, 0.25) | 0.37 (0.32, 0.41) |
| Integrated Gradients | 0.17 (0.13, 0.20) | 0.21 (0.18, 0.25) | 0.37 (0.33, 0.41) |
| Guided Backpropagation | 0.36 (0.30, 0.42) | 0.18 (0.14, 0.22) | 0.31 (0.25, 0.37) |
| LRP | 0.20 (0.16, 0.24) | 0.21 (0.17, 0.25) | 0.33 (0.28, 0.37) |
| Grad-CAM | 0.21 (0.17, 0.26) | 0.31 (0.26, 0.36) | 0.38 (0.33, 0.43) |
| DeconvNet | 0.16 (0.13, 0.20) | 0.17 (0.13, 0.21) | 0.25 (0.20, 0.30) |
| SmoothGrad | 0.21 (0.18, 0.25) | 0.26 (0.22, 0.30) | 0.38 (0.33, 0.43) |
| SmoothGrad-SQ | 0.19 (0.15, 0.23) | 0.27 (0.23, 0.30) | 0.32 (0.29, 0.36) |
| VarGrad | 0.17 (0.13, 0.20) | 0.27 (0.24, 0.31) | 0.24 (0.20, 0.28) |
| Baseline | 0.12 (0.11, 0.13) | 0.12 (0.11, 0.13) | 0.12 (0.11, 0.13) |

*Table E.3 Average ECS on the IDRiD training set and 95% confidence intervals, for hemorrhages only.*

|  | ResNet50 | InceptionV3 | VGG16 |
| --- | --- | --- | --- |
| Gradients | 0.12 (0.07, 0.17) | 0.10 (0.05, 0.15) | 0.21 (0.13, 0.28) |
| Input * Gradients | 0.13 (0.08, 0.18) | 0.14 (0.08, 0.19) | 0.23 (0.15, 0.30) |
| Integrated Gradients | 0.10 (0.06, 0.15) | 0.10 (0.05, 0.15) | 0.22 (0.14, 0.30) |
| Guided Backpropagation | 0.14 (0.08, 0.20) | 0.09 (0.04, 0.14) | 0.20 (0.13, 0.28) |
| LRP | 0.08 (0.02, 0.14) | 0.14 (0.08, 0.19) | 0.16 (0.09, 0.23) |
| Grad-CAM | 0.12 (0.07, 0.17) | 0.14 (0.08, 0.21) | 0.19 (0.12, 0.26) |
| DeconvNet | 0.09 (0.04, 0.14) | 0.09 (0.04, 0.15) | 0.17 (0.10, 0.24) |
| SmoothGrad | 0.29 (0.18, 0.41) | 0.17 (0.09, 0.24) | 0.22 (0.14, 0.30) |
| SmoothGrad-SQ | 0.28 (0.17, 0.39) | 0.15 (0.09, 0.21) | 0.11 (0.06, 0.15) |
| VarGrad | 0.22 (0.14, 0.31) | 0.16 (0.09, 0.23) | 0.08 (0.04, 0.13) |
| Baseline | 0.07 (0.05, 0.09) | 0.07 (0.05, 0.09) | 0.07 (0.05, 0.09) |

*Table E.4 Average ECS on the IDRiD training set and 95% confidence intervals, for soft exudates only.*
